# Necrotising Fasciitis: A 10-Year Retrospective Cohort of Outcomes and Correlation to the Laboratory Risk Indicator for Necrotising Fasciitis (LRINEC) Score in a Single-Centre District General Hospital in the United Kingdom

**DOI:** 10.7759/cureus.108335

**Published:** 2026-05-06

**Authors:** Spilios Dellis, Thomas L Lewis, Stavros Tsotsolis, Chigoziem A Ogbolu, Charlotte Larkin, Kamalpreet S Cheema

**Affiliations:** 1 School of Physical Education and Sports Science, National and Kapodistrian University of Athens, Athens, GRC; 2 Trauma and Orthopaedic Surgery, King’s College Hospital NHS Foundation Trust, London, GBR; 3 Trauma and Orthopaedics, Lewisham and Greenwich NHS Trust, London, GBR; 4 Faculty of Medicine, King’s College London, London, GBR; 5 Trauma and Orthopaedic Surgery, Lewisham and Greenwich NHS Trust, London, GBR

**Keywords:** icu admission, lrinec, mortality, necrotising fasciitis, soft-tissue infection, surgical debridement

## Abstract

Background

Necrotising fasciitis (NF) is a life-threatening soft-tissue infection associated with high morbidity and mortality. The Laboratory Risk Indicator for Necrotising Fasciitis (LRINEC) score was developed to aid early diagnosis, but its association with clinical outcomes remains uncertain. Most existing data originate from tertiary referral centres. This study evaluated clinical outcome, LRINEC score performance, and surgical timing in NF cases managed in a district general hospital in the United Kingdom over a 10-year period.

Methodology

This retrospective cohort study followed STROBE guidelines and included 48 adult patients with surgically confirmed NF (clinical features, laboratory parameters, intraoperative findings, and/or histopathological confirmation) treated between January 2013 and December 2023. Variables collected included demographics, comorbidities, LRINEC scores, time to first debridement, number of surgical debridements, intensive care unit (ICU) admission, hospital length of stay, and in-hospital mortality. Statistical analysis included chi-square or Fisher’s exact tests, non-parametric comparisons (Kruskal-Wallis test, Mann-Whitney U test), and Spearman’s correlation analyses, with statistical significance set at p-values <0.05. Owing to sample size, analyses were exploratory and unadjusted; multivariable modelling was not performed. The study was registered and approved as a Clinical Audit by the Trust Clinical Audit Review Panel (reference: 7926). Individual consent was waived as anonymised retrospective audit data were used.

Results

The mean patient age was 62.2 years (SD = 12.1), with 56.2% male predominance (27/48). Lower limbs were affected in 83.3% (40/48) of cases. Mean LRINEC score was 7.9 ± 2.3, with 66.7% (32/48) classified as high-risk (≥8). Overall mortality was 27.1% (13/48), and 81% (39/48) required ICU admission. Mean hospital stay was 24.3 ± 18.6 days; patients required an average of 3.0 ± 1.4 surgical debridements. Group A *Streptococcus* was the most common pathogen (21/48, 43.8%). Higher LRINEC scores showed a positive correlation with the number of surgical debridements required (Spearman’s ρ = 0.32, p = 0.027) but not with mortality or ICU admission. Shorter time to surgery was associated with higher mortality (p = 0.031), which may reflect confounding by indication in this unadjusted analysis.

Conclusions

In district hospital settings, NF carries substantial morbidity and mortality despite aggressive management. In this cohort, higher LRINEC scores were associated with a greater number of surgical debridements but not with mortality. The inverse relationship between shorter time to surgery and mortality likely reflects confounding by indication, though this could not be confirmed in this unadjusted analysis. These exploratory findings suggest that LRINEC may have a role in anticipating operative demand, but larger prospective studies are needed to validate this observation.

## Introduction

Necrotising fasciitis (NF) is a rare, life-threatening bacterial soft-tissue infection characterised by rapid spread along fascial planes and necrosis of both fascia and surrounding soft tissues [1]. Infection is most often polymicrobial or caused by β-haemolytic Group A *Streptococcus* [1]. This devastating soft-tissue infection is associated with significant morbidity and mortality, with reported rates ranging from 10% to 76% across published series [2,3]. Despite advances in recognition and management, mortality rates remain substantial [4-6].

Prompt recognition, aggressive serial debridement, broad-spectrum antibiotics, and coordinated critical-care management are essential for favourable outcomes [7]. Delays in diagnosis or surgical intervention have been associated with increased morbidity and mortality [7,8]. The Laboratory Risk Indicator for Necrotising Fasciitis (LRINEC) score, based on routine laboratory parameters, was developed to aid early diagnosis and help distinguish NF from other soft-tissue infections [9]. However, its association with clinical outcomes in patients with NF has not been well established, with studies producing conflicting results [10-13].

Existing literature predominantly originates from tertiary or regional referral centres [5,6,14], where resource availability and patient profiles differ from those encountered in district general hospital settings. Data on outcomes of NF managed in community hospital environments remain limited.

This study examined outcomes of adult patients with NF diagnosed based on clinical, intraoperative, and/or histopathological findings, treated at a district general hospital in the United Kingdom over a 10-year period. The study explored the association of LRINEC score and time to surgery with in-hospital outcomes, including mortality, ICU admission, and the number of surgical debridements required.

## Materials and methods

Participants and variables

This retrospective cohort study was conducted in accordance with the STROBE guidelines for reporting observational studies [15]. Potential cases were identified from hospital electronic records using International Statistical Classification of Diseases and Related Health Problems, 10th Revision (ICD-10) code M72.6 (‘necrotising fasciitis’) between January 2013 and December 2023. Of 64 records screened, 16 were excluded: two with incomplete admission laboratory data, seven without histopathological confirmation, and seven reclassified as deep cellulitis without fascial involvement, yielding a final cohort of 48 patients. The time of presentation was defined as the time of triage at the emergency department. Variables collected included demographics, comorbidities, laboratory values (for LRINEC calculation), imaging when performed, operative details, ICU admission and duration, hospital length of stay, and in-hospital mortality.

All 48 patients underwent surgical debridement, and diagnosis of NF was confirmed intraoperatively by characteristic findings (grey necrotic fascia, lack of tissue adherence, absence of bleeding) and by histopathological examination of debrided tissue in all cases. The LRINEC score was calculated from routine admission laboratory values (C-reactive protein, white blood cell count, haemoglobin, sodium, creatinine, and glucose), which were available for all patients at presentation. LRINEC scores were complete for the entire cohort. For the purposes of this study, the LRINEC score was analysed as an exposure variable to explore its association with clinical outcomes, independent of its role in initial clinical assessment. Scores ≥8 indicated high risk (Table 1).

**Table 1:** Diagnostic criteria for necrotising fasciitis. CRP = C-reactive protein; WCC = white cell count

Diagnostic category	Findings
Clinical signs	Rapidly spreading swelling and erythema, severe pain disproportionate to clinical findings, clinical features of sepsis
Laboratory findings	Raised CRP, elevated WCC, low haemoglobin, hyponatraemia, raised creatinine, hyperglycaemia
Intraoperative findings	Grey necrotic fascia, minimal bleeding, little resistance to blunt dissection, foul-smelling ‘dishwater’ pus
Histopathological findings	Deep fascial necrosis, intense neutrophilic infiltration, polymicrobial infection

Inclusion and exclusion criteria

**Figure 1:**
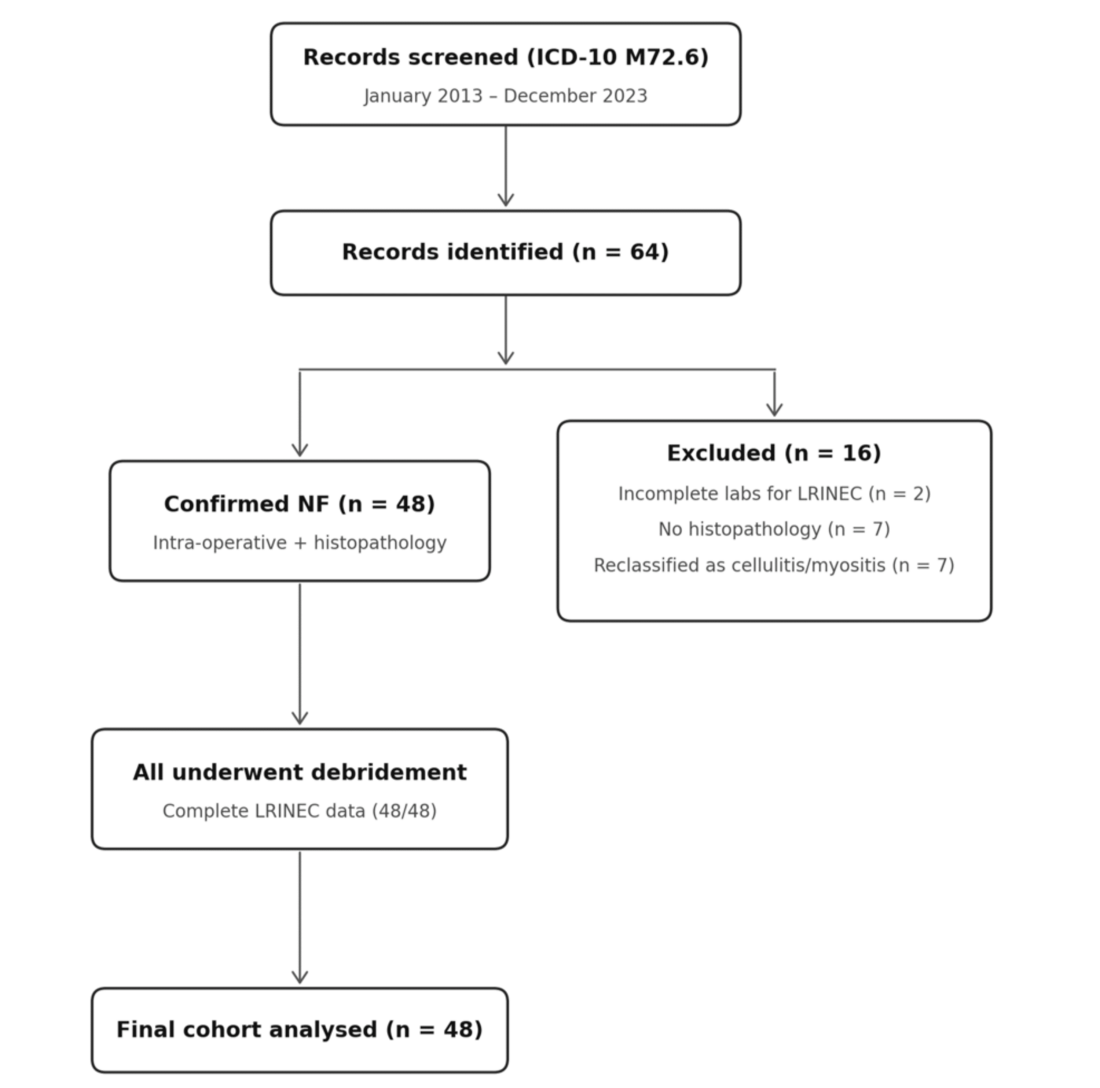
Patient flow diagram showing screening, exclusion criteria, and final cohort selection (STROBE). ICD-10 = International Statistical Classification of Diseases and Related Health Problems, 10th Revision; NF = necrotising fasciitis; LRINEC = Laboratory Risk Indicator for Necrotising Fasciitis

All consecutive patients aged ≥18 years identified by ICD-10 code M72.6 between January 2013 and December 2023 were screened. Inclusion required intraoperative confirmation of NF (characteristic findings of fascial necrosis), histopathological confirmation from debrided tissue, and complete admission laboratory values for LRINEC calculation. Of 64 records screened, 16 were excluded: two with incomplete admission laboratory data precluding LRINEC calculation, seven without histopathological confirmation, and seven reclassified as deep cellulitis or myositis without fascial involvement on operative and histopathological review. The final cohort comprised 48 patients. All patients presented directly through the emergency department; none were transferred from other institutions. No patient had a recurrent episode, and no patient died before surgical intervention. The patient flow diagram is provided in Figure 1.

Primary outcomes were in-hospital mortality, ICU admission, length of hospital stay, and number of surgical debridements. The primary exposures were the LRINEC score, calculated from routine admission laboratory values and analysed independently of its role in initial clinical assessment, and time from emergency department triage to first surgical debridement (hours). Demographic variables collected included age, sex, and site of infection (upper vs. lower limb). Comorbidities (including obesity, smoking, diabetes, hypertension, COPD, malignancy, alcohol dependence, immunosuppression, and chronic kidney disease) were extracted retrospectively from clinical records.

Bias

All consecutive cases (2013-2023) were included using predefined clinical, operative, and histopathological criteria to reduce selection and misclassification bias. However, several sources of bias should be acknowledged. Exclusion of 16 patients with incomplete records may have introduced selection bias, particularly if data completeness was associated with disease severity or outcome. Case identification via ICD-10 coding is subject to misclassification bias, although all included cases were subsequently confirmed intraoperatively and histopathologically. As a retrospective chart review, information bias is inherent, particularly for timing variables (e.g. time from triage to surgery) and severity assessments, which rely on the accuracy and completeness of contemporaneous clinical documentation. We anticipated potential confounding by indication, as patients presenting in a more critical condition were prioritised for immediate surgical intervention. As multivariable modelling was not performed owing to the small sample size, associations were interpreted in the context of baseline severity and should be considered unadjusted.

Study size

All eligible cases during 2013-2023 were included; no prior sample size calculation was performed. This is a small exploratory cohort with limited statistical power. The sample size constrains the ability to detect meaningful differences in outcomes such as mortality, particularly when stratified into LRINEC risk categories and surgical timing subgroups, and estimates within these subgroups may be unstable. Multivariable modelling was not performed, and subgroup analyses should be interpreted with caution.

Statistical methods

Categorical outcomes (mortality by LRINEC risk category, mortality by timing group, mortality by organism type) were analysed using chi-square or Fisher’s exact tests. Comparisons of continuous or ordinal variables across three or more groups (number of debridements by LRINEC risk category, number of debridements by timing group) used the Kruskal-Wallis test. Two-group comparisons of continuous data (hospital stay in survivors vs. non-survivors) used the Mann-Whitney U test. Correlations between continuous variables (LRINEC score vs. number of debridements, time to surgery vs. mortality, LRINEC score vs. hospital stay) were assessed using Spearman’s rank correlation coefficient. Odds ratios (ORs) with 95% confidence intervals (CIs) were calculated for binary outcome comparisons (early vs. delayed surgery mortality). Statistical significance was set at α = 0.05.

All reported associations are unadjusted. Multivariable modelling was not performed owing to the small sample size. This is a small exploratory cohort with limited statistical power, wide CIs, and a risk of imprecise estimates, particularly in subgroup analyses. Multiple comparisons were performed without formal correction, increasing the possibility of type I error. Findings should, therefore, be interpreted as hypothesis-generating rather than confirmatory.

Ethical approval

This study was registered and approved as a Clinical Audit by the local NHS Trust Clinical Audit Review Panel (project number: 7926). As anonymised retrospective audit data were used, individual patient consent was waived. Data were handled in compliance with UK General Data Protection Regulation (GDPR) requirements.

## Results

Demographics

A total of 48 patients were treated for NF at our institution between 2013 and 2023. The mean age was 62.2 years (SD = 12.1; range = 34-84). There was a male predominance with 56.2% male patients (n = 27) and 43.8% female patients (n = 21). The lower limbs were affected in 40 (83.3%) cases, and the upper limbs in 8 (16.7%).

Comorbidities

Comorbidities were present in 45/48 (93.8%) patients, with a mean of 2.8 comorbidities per affected patient. The full comorbidity profile is presented in Table 2. The most prevalent conditions were obesity (36/48, 75.0%) and smoking (29/48, 60.4%), followed by hypertension and diabetes mellitus (each 14/48, 29.2%).

**Table 2:** Comorbidity profile of patients with necrotising fasciitis (N = 48). Comorbidities were present in 45/48 (93.8%) patients, with a mean of 2.8 per affected patient. Patients may have more than one comorbidity. COPD = chronic obstructive pulmonary disease

Comorbidity	n	%
Obesity	36	75.0
Smoking	29	60.4
Hypertension	14	29.2
Diabetes mellitus	14	29.2
Cancer	7	14.6
COPD	7	14.6
Alcohol dependence	7	14.6
Immunosuppression	6	12.5
Chronic kidney disease	6	12.5

Clinical presentation

During the initial presentation, 27/48 (56.3%) had pyrexia (>38°C), and tachycardia (>100 beats/minute) was observed in 31/48 (64.6%). Hypotension was present in 19/48 (39.6%). All patients presented with pain and swelling, and erythema was observed in 46/48 (95.8%). Common clinical features are summarised in Table 3.

**Table 3:** Clinical presentation at admission.

Clinical feature	n (%)
Pain	48 (100)
Swelling	48 (100)
Erythema	46 (95.8)
Tachycardia (>100 beats/minute)	31 (64.6)
Pyrexia (>38°C)	27 (56.3)
Hypotension	19 (39.6)

Causative organisms

**Figure 2:**
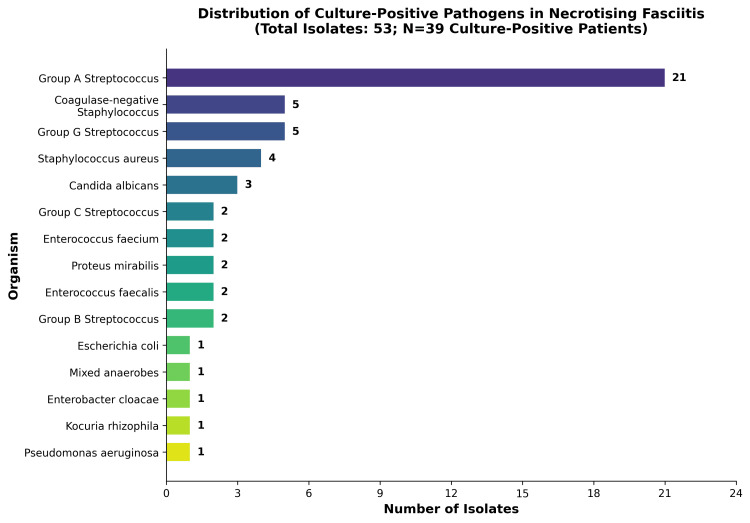
Distribution of culture-positive pathogens in necrotising fasciitis.

Cultures were positive in 39/48 patients (81.2%), while 9/48 (18.8%) were culture-negative. Fifteen unique pathogens were isolated. Group A *Streptococcus* (GAS) predominated (21/48, 43.8%), and 12/48 (25.0%) of cases were polymicrobial; all polymicrobial infections involved GAS alongside other aerobic and/or anaerobic organisms. Among GAS-positive patients, mortality was higher in polymicrobial infections (≈38%) than in monomicrobial GAS infections (≈16%) (χ² test, used to compare categorical proportions between organism groups, p = 0.030). Among culture-negative patients, mortality was 3/9 (33.3%) compared with 10/39 (25.6%) in culture-positive patients. The distribution of culture results is depicted in Figure 2.

LRINEC scoring

The cohort had a mean LRINEC score of 7.96 ± 2.32 (median = 8.0, interquartile range (IQR) = 7.0-9.0). Stratification by risk category showed 32/48 (66.7%) as high risk (≥8), 10/48 (20.8%) as intermediate risk (6-7), and 6/48 (12.5%) as low risk (<6). Mortality rates across risk categories were 12/32 (37.5%) in high-risk, 0/10 (0%) in intermediate-risk, and 1/6 (16.7%) in low-risk patients; this trend did not reach statistical significance (χ² test, used to compare categorical proportions across risk groups, p = 0.055). When LRINEC risk categories were compared by number of debridements, no significant difference was found (Kruskal-Wallis test, used to compare distributions across three independent groups, H = 0.77, p = 0.680). Hospital and ICU durations were similar across risk strata.

**Figure 3:**
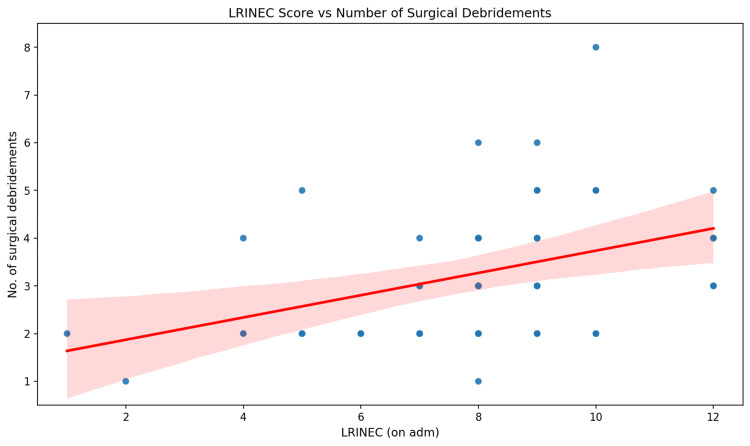
Correlation between LRINEC scores and number of surgical debridements. LRINEC = Laboratory Risk Indicator for Necrotising Fasciitis

**Figure 4:**
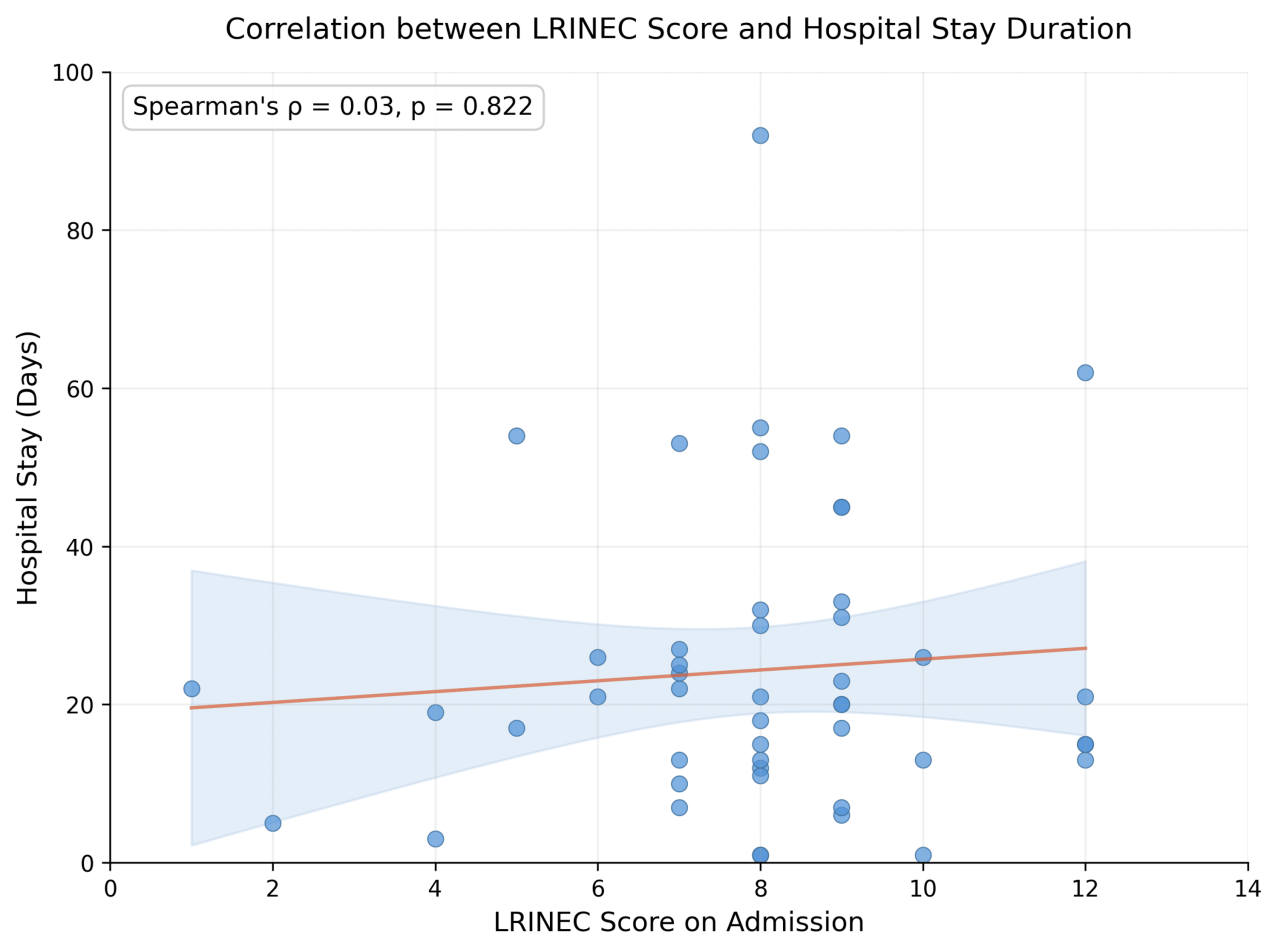
Correlation between LRINEC scores and hospital stay duration. LRINEC = Laboratory Risk Indicator for Necrotising Fasciitis

When analysed as a continuous variable, higher LRINEC scores showed a modest positive correlation with the number of surgical debridements (Spearman’s rank correlation, used as both variables were non-normally distributed, ρ = 0.32, p = 0.027). However, Figure 3 demonstrates substantial scatter around this trend, indicating considerable individual variation. The discrepancy between the non-significant grouped analysis (p = 0.680) and the significant continuous-score correlation (p = 0.027) may reflect loss of statistical information when continuous scores are categorised into risk groups, particularly in a small sample. LRINEC scores showed minimal correlation with hospital stay duration (Spearman’s ρ = 0.03, p = 0.822), with considerable dispersion as shown in Figure 4. This exploratory association between continuous LRINEC scores and the number of debridements is unadjusted and should be interpreted with caution, given the small sample size, weak effect, and absence of multivariable modelling.

Time to surgery

**Figure 5:**
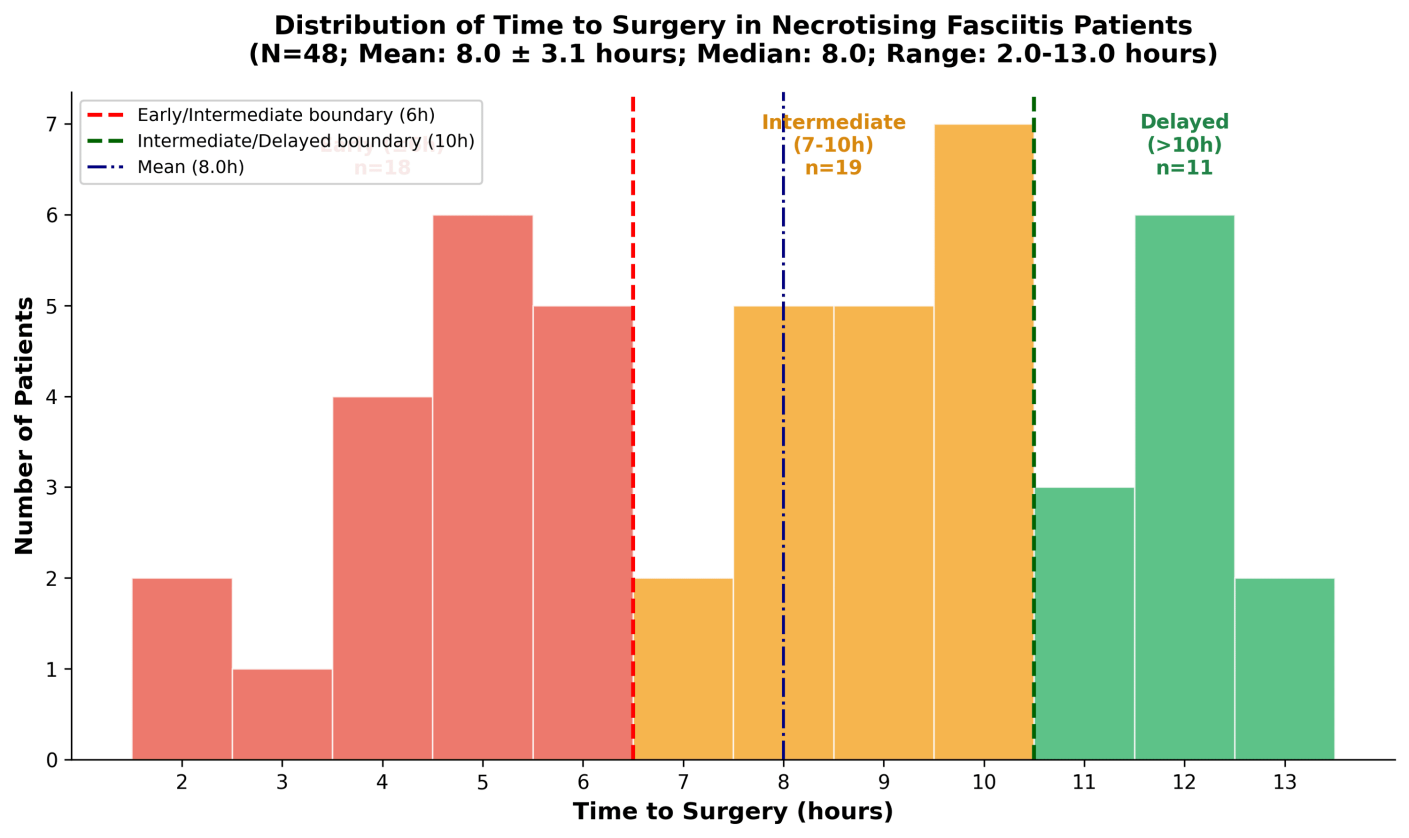
Distribution of time to surgery.

The mean time from emergency department triage to first surgical debridement was 8.0 ± 3.1 hours (median = 8.0, range = 2-13). To explore the relationship between surgical timing and outcomes, patients were stratified into the following three groups based on the observed data distribution: early (≤6 hours, n = 18), intermediate (7-10 hours, n = 19), and delayed (>10 hours, n = 11). These thresholds were selected to approximate tertiles of the cohort and reflect natural groupings in the distribution (Figure 5). This stratification was exploratory and not based on established clinical cutoffs.

There was a negative correlation between time from emergency department triage to first surgical debridement and mortality (Spearman’s rank correlation, used as the outcome was ordinal, ρ = -0.31, p = 0.031, 95% CI = -0.547 to -0.029). Patients receiving early surgery (≤6 hours) had higher odds of mortality compared with those operated after 6 hours (intermediate + delayed) (OR = 6.50, 95% CI = 1.60-26.38, p = 0.015). The number of debridements, hospital stay, and ICU duration did not differ significantly between timing groups.

In this unadjusted analysis, shorter time to surgery was associated with higher mortality rates. One possible explanation is confounding by indication, whereby the most critically unwell patients were prioritised for immediate surgery. However, as no adjusted analysis was performed, this interpretation remains inferential and cannot be confirmed from these data. This finding should not be interpreted as evidence against early surgical intervention.

Number of debridements

All 48 patients underwent at least one surgical debridement, consisting of excision of necrotic tissue to healthy bleeding borders. Four (4/48, 8.3%) patients died within 48 hours of their initial debridement and therefore did not undergo further procedures; this early mortality may result in an underestimation of the procedural burden that would otherwise have been required. Among the remaining 44 patients, repeat debridements were performed as clinically indicated. The mean number of surgical debridements was 3.0 ± 1.4 (median = 2.0, range = 1-8): 24/48 (50.0%) required two debridements, 10/48 (20.8%) required three, 9/48 (18.8%) required four, and 1/48 (2.1%) required eight, representing the maximum in this series. The number of debridements did not differ significantly between timing groups (Kruskal-Wallis test, used to compare distributions across three independent groups, H = 1.748, p = 0.417) and showed no correlation with mortality (Spearman's ρ = 0.005, p = 0.971). However, the number of debridements is inherently influenced by survival time, as patients who survive longer have more opportunity for repeat procedures. In this unadjusted analysis, the relationship between the number of debridements and clinical outcomes should be interpreted with caution.

Mortality and duration of hospital stay

**Figure 6:**
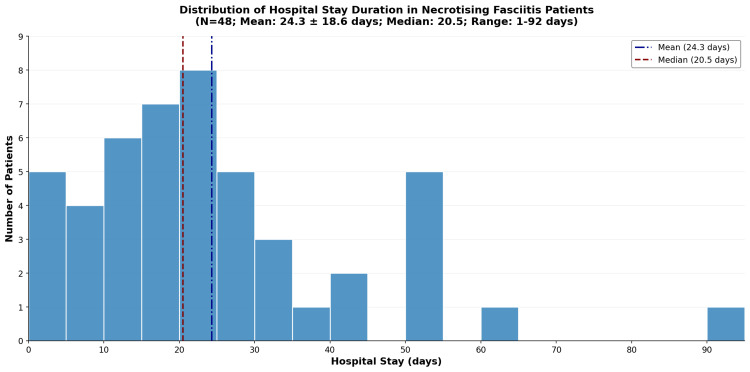
Distribution of hospital stay duration.

Overall mortality in this cohort was 27.1% (13/48; 95% CI = 15.3-41.4%). Hospital stay duration ranged from 1 to 92 days with a mean of 24.3 ± 18.6 days (median = 20.5 days, IQR = 13.0-30.2 days). Survivors had a longer mean hospital stay compared to non-survivors (26.6 ± 19.3 days vs. 18.3 ± 15.9 days), though this difference was not statistically significant (Mann-Whitney U test, used for two-group comparison of non-normally distributed continuous data, U = 279.5, p = 0.232, effect size r = 0.174). The distribution of hospital stay duration is shown in Figure 6.

Mortality rates differed by timing of initial surgical debridement (χ² test, used to compare categorical proportions across timing groups, χ² = 7.817, df = 2, p = 0.020). Patients in the early surgery group (≤6 hours) had a mortality rate of 50.0% (9/18, 95% CI = 27.8-72.2%), compared with 15.8% (3/19, 95% CI = 0.0-31.6%) in the intermediate group (7-10 hours) and 9.1% (1/11, 95% CI = 0.0-27.3%) in the delayed group (>10 hours). This association is unadjusted and may be influenced by confounding by indication; interpretation is discussed further in the Discussion section.

There was no correlation between time from emergency department triage to first surgical debridement and hospital stay duration (Spearman's rank correlation, ρ = -0.001, p = 0.995). A summary of all statistical analyses performed in this study is presented in Table 4.

**Table 4:** Summary of statistical analyses. Statistical significance was set at p-values <0.05. *: denotes statistically significant result (p < 0.05). All analyses are unadjusted and exploratory. χ² = chi-squared; ρ = Spearman’s rank correlation coefficient; OR = odds ratio; CI = confidence interval; df = degrees of freedom; r = effect size; GAS = Group A *Streptococcus*; LRINEC = Laboratory Risk Indicator for Necrotising Fasciitis

Analysis	Observed data	Test	Statistic	df	P-value	Effect size/CI
LRINEC risk category vs. mortality	High: 12/32 (37.5%); Intermediate: 0/10 (0%); Low: 1/6 (16.7%)	χ² test (trend)	χ² = 5.82	2	0.055	—
LRINEC score vs. number of debridements	Continuous variables (n = 48)	Spearman’s ρ	ρ = 0.32	—	0.027*	—
LRINEC risk group vs. number of debridements	High/Intermediate/Low risk groups (n = 48)	Kruskal-Wallis	H = 0.77	2	0.680	—
LRINEC score vs. hospital stay	Continuous variables (n = 48)	Spearman’s ρ	ρ = 0.03	—	0.822	—
Time to surgery vs. mortality	Continuous variables (n = 48)	Spearman’s ρ	ρ = −0.31	—	0.031*	95% CI = −0.547 to −0.029
Early (≤6 hours) vs. >6 hours mortality	Early: 9/18 (50.0%) >6 hours: 4/30 (13.3%)	Odds Ratio	OR = 6.50	—	0.015*	95% CI = 1.60–26.38
Mortality by timing group	Early: 9/18 (50.0%); Intermediate: 3/19 (15.8%); Delayed: 1/11 (9.1%)	χ² test	χ² = 7.817	2	0.020*	—
Mortality by organism type	Mixed GAS: ≈38% GAS-only: ≈16%	χ² test	χ² = 4.71	1	0.030*	—
Number of debridements vs. timing group	Early/Intermediate/Delayed groups (n = 48)	Kruskal-Wallis	H = 1.748	2	0.417	—
Number of debridements vs. mortality	Continuous variables (n = 48)	Spearman’s ρ	ρ = 0.005	—	0.971	—
Hospital stay: Survivors vs. non-survivors	Survivors: 26.6 ± 19.3 days; Non-survivors: 18.3 ± 15.9 days	Mann-Whitney U	U = 279.5	—	0.232	r = 0.174
Time to surgery vs. hospital stay	Continuous variables (n = 48)	Spearman’s ρ	ρ = −0.001	—	0.995	—

## Discussion

Main findings

In this retrospective cohort of 48 patients with NF managed at a single district general hospital in the United Kingdom over a 10-year period, patients required a mean of 3.0 surgical debridements and a mean hospital stay of 24.3 days. Overall mortality was 27.1% (13/48), and 81% (39/48) required ICU admission. Comorbidities were present in 93.8% (45/48), with a mean of 2.8 per affected patient. There was a male predominance (56.2% vs. 43.8%) and lower limb involvement in 83.3% of cases.

The key exploratory findings of this study were: (1) a modest positive correlation between continuous LRINEC scores and the number of surgical debridements (Spearman’s ρ = 0.32, p = 0.027), though this was not reflected when LRINEC was analysed by risk category (p = 0.680); (2) an association between shorter time to surgery and higher mortality (ρ = −0.31, p = 0.031), which may reflect confounding by indication but could not be confirmed in this unadjusted analysis; and (3) no significant association between LRINEC scores and mortality (p = 0.055) or hospital stay (ρ = 0.03, p = 0.822).

Although early surgical debridement is universally advocated in NF [7], patients in the early surgery group (≤6 hours) in this cohort had the highest mortality rate (50.0%). This association may reflect confounding by indication, whereby patients presenting in the most critical physiological state were prioritised for immediate intervention. However, as no adjusted analysis was performed, this interpretation remains inferential and cannot be confirmed from these data. The observed inverse relationship between shorter time to surgery and mortality should not be interpreted as evidence against early surgical intervention. Prospective studies with adjustment for baseline physiological severity would be needed to clarify the relationship between surgical timing and outcomes in NF.

Comorbidities were prevalent in this cohort (93.8%), and lower limb involvement predominated (83.3%), consistent with published series [5,6]. LRINEC scores were not significantly associated with mortality across risk categories (p = 0.055) or with ICU admission. When analysed as a continuous variable, LRINEC showed a modest correlation with the number of debridements (ρ = 0.32, p = 0.027), but this was not replicated in the grouped analysis (p = 0.680), and the clinical significance of this weak association is uncertain. These findings do not support the use of LRINEC as a prognostic tool, which is consistent with its original development as a diagnostic aid rather than an outcome predictor [9]. Whether the observed association with the number of debridements reflects a true relationship with disease extent cannot be determined from this unadjusted exploratory analysis.

Patient demographics and comorbidity burden

The demographic profile of this cohort, i.e., older age (mean 62.2 years), male predominance (56.2%), and lower limb involvement (83.3%), is consistent with findings reported by Bilolikar et al. and Guliyeva et al. in contemporary NF series [5,6]. Comorbidity burden was high: 45/48 (93.8%) had at least one comorbidity, with a mean of 2.8 per affected patient. Obesity was the most prevalent condition (75.0%), followed by smoking (60.4%), hypertension (29.2%), and diabetes mellitus (29.2%). This comorbidity profile is notable for the high prevalence of obesity, which has been increasingly recognised as a risk factor for NF and may contribute to delayed clinical recognition due to deeper soft-tissue involvement [6]. The high rates of diabetes and hypertension are consistent with established risk factors reported across multiple NF cohorts [5,6,14]. However, the relationship between individual comorbidities and clinical outcomes (mortality, ICU admission, number of debridements) was not formally tested in this study, and whether comorbidity burden influenced outcomes in this cohort cannot be determined. This represents a limitation and an area for future investigation in larger cohorts where multivariable analysis would be feasible.

Pathogens

Pathogens identified in NF can vary, and this is commonly a polymicrobial infection [14]. Positive cultures were obtained in 39/48 (81.2%) of cases. GAS was the most frequent isolate (21/48, 43.8%), followed by polymicrobial infections (12/48, 25.0%), all of which involved GAS alongside other aerobic and/or anaerobic organisms. Mortality was higher in polymicrobial infections involving GAS (≈38%) than in monomicrobial GAS infections (≈16%), a difference that reached statistical significance (χ² = 4.71, p = 0.030). Culture-negative patients had a mortality rate of 33.3% (3/9) compared with 25.6% (10/39) in culture-positive patients. The overall microbiological spectrum is broadly consistent with contemporary UK and international data [14,16] and supports the use of broad-spectrum empiric antimicrobial therapy, with early microbiology consultation and prompt culture-guided de-escalation.

LRINEC score performance

NF is clinically challenging to differentiate from severe cellulitis in the early stages. In 2004, Wong et al. developed the LRINEC score based on six laboratory parameters to aid early diagnosis and help distinguish NF from other soft-tissue infections [9]. While the initial study reported excellent predictive value, subsequent validation studies have demonstrated high false-negative rates, low sensitivity, and poor specificity [10-13].

In this cohort, LRINEC scores were not significantly associated with mortality when analysed by risk category (p = 0.055). When analysed as a continuous variable, LRINEC showed a modest correlation with the number of debridements (ρ = 0.32, p = 0.027), but this was not replicated in the grouped analysis (p = 0.680). This discrepancy may reflect loss of statistical power when continuous scores are categorised into risk groups in a small sample, but could also indicate that the continuous-score association is fragile. LRINEC showed no meaningful correlation with hospital stay (ρ = 0.03, p = 0.822). These findings are consistent with the broader literature suggesting variable diagnostic and limited prognostic performance of the LRINEC score [13-16]. At most, this exploratory analysis suggests a cautious association between LRINEC scores and the number of debridements, but this is unadjusted, modest in effect size, and insufficient to establish LRINEC in any prognostic, severity, or resource-planning role. LRINEC remains, as originally intended, a diagnostic aid [9].

Mortality and duration of hospital stay

The overall mortality rate in this cohort was 27.1% (13/48). This falls within the range reported in the literature, which varies from approximately 10% to 76% depending on study populations, disease severity, pathogens, and management protocols [2,3,6]. Our mortality rate is comparable to that reported in other cohorts employing aggressive surgical management, including Bucca et al. (8.3% with early debridement) [3] and Glass et al. (20.8% in a UK metropolitan population) [16].

Survivors had a longer mean hospital stay than non-survivors (26.6 vs. 18.3 days), though this difference was not statistically significant (Mann-Whitney U = 279.5, p = 0.232). One possible explanation is that survivors had more time to undergo repeated debridements, wound care, and rehabilitation, whereas non-survivors had truncated hospital stays due to early death. However, this was not formally analysed and remains speculative.

Surgical management and patient outcomes

Confirmation of NF diagnosis is frequently intraoperative, based on characteristic findings such as fascial necrosis, lack of tissue adherence, and absence of bleeding [7,14]. Early and thorough surgical debridement to healthy bleeding tissue remains the cornerstone of management [7,8]. In this cohort, patients required a mean of 3.0 surgical debridements (range = 1-8), consistent with published data reporting the need for serial procedures to achieve source control [14]. Four (8.3%) patients died within 48 hours of their initial debridement and did not undergo further procedures, which may underestimate the procedural burden that would otherwise have been required. The number of debridements was not significantly associated with mortality (ρ = 0.005, p = 0.971) or with timing group (p = 0.417), though as discussed above, survival time inherently influences the opportunity for repeat procedures.

Patients with NF often require prolonged hospital stays and ICU admissions due to the severity of their condition. Hospital stay has been reported to range from 15 to 60 days, with the need for multiple surgeries and critical care support [14]. In our cohort, the mean hospital stay was 24 days. Additionally, up to two-thirds of patients with NF have been reported to require admission to the ICU. ICU admission rate was 81%, consistent with previous UK data [14,16].

Limitations and future directions

This was a small, exploratory, retrospective single-centre cohort study, and several important limitations should be acknowledged. All reported associations are unadjusted and therefore highly vulnerable to confounding, particularly by baseline physiological severity, which could not be accounted for without multivariable modelling. The sample size (n = 48) limits statistical power, particularly for subgroup analyses across LRINEC risk categories and timing groups, where small denominators produce wide CIs and imprecise estimates. Exclusion of 16 patients with incomplete records may have introduced selection bias, particularly if data completeness was associated with disease severity or outcome. As a retrospective chart review, information bias is inherent: timing variables, clinical assessments, and comorbidity recording rely on the accuracy of contemporaneous documentation. Residual confounding cannot be ruled out due to the absence of multivariable modelling.

Despite these limitations, this cohort provides descriptive data on necrotising fasciitis outcomes in a district general hospital setting in the United Kingdom, where published data remain limited. The findings should be considered hypothesis-generating rather than confirmatory. Larger, prospective multicentre studies with adjustment for baseline severity and comorbidity burden are needed to clarify the associations observed in this analysis, particularly the relationship between LRINEC scores and number of debridements, and between surgical timing and mortality.

Clinical implications

Several observations from this cohort may be relevant to clinical practice, though they should be interpreted cautiously given the study’s exploratory nature and limitations. The high ICU admission rate (81%) is consistent with published data [3,16] and suggests that early critical care involvement should be considered in suspected NF cases presenting to district general hospitals. The observed association between shorter time to surgery and higher mortality, while likely reflecting confounding by indication rather than harm from early intervention, serves as a reminder that surgical timing data in NF should be interpreted alongside baseline physiological status. The modest exploratory association between LRINEC scores and the number of debridements (ρ = 0.32, p = 0.027) requires validation in larger adjusted studies before any role in clinical decision-making can be considered. This cohort demonstrates that NF can be managed in a district general hospital setting with mortality rates within the range reported in the wider literature [2,3,6], though direct comparison with tertiary centre outcomes was not performed, and no conclusions about optimal care setting can be drawn from these data.

## Conclusions

This retrospective single-centre study describes outcomes of 48 patients with NF managed at a district general hospital in the United Kingdom over 10 years. Mortality was 27.1%, ICU admission was required in 81%, and patients underwent a mean of 3.0 surgical debridements, with a mean hospital stay of 24.3 days. A modest exploratory association was observed between higher continuous LRINEC scores and the number of debridements, though this was not replicated in grouped analysis, and its clinical significance is uncertain. Shorter time to surgery was associated with higher mortality in this unadjusted analysis, which may reflect confounding by indication but cannot be confirmed without adjusted modelling. LRINEC scores were not significantly associated with mortality. Larger prospective multicentre studies with multivariable adjustment are needed to clarify these associations.
